# Orbital positron emission tomography/computed tomography (PET/CT) imaging findings in graves ophthalmopathy

**DOI:** 10.1186/1756-0500-6-353

**Published:** 2013-09-04

**Authors:** Leonardo García-Rojas, Gloria Adame-Ocampo, Guillermo Mendoza-Vázquez, Erick Alexánderson, José Luis Tovilla-Canales

**Affiliations:** 1 Instituto de Oftalmología Fundación “Conde de Valenciana” I.A.P., Mexico City, Mexico; 2 Unidad PET/CT Ciclotrón, Facultad de Medicina, Universidad Nacional Autónoma de México, Mexico City, Mexico

## Abstract

**Background:**

We aimed to describe orbital positron emission tomography/computed tomography (PET/CT) imaging findings, both structural and metabolic, in different clinical stages of Graves ophthalmopathy (GO). This prospective, observational, cross-sectional study examined 32 eyes of 16 patients with GO.

**Methods:**

Patients were assessed with a complete ophthalmological evaluation and assigned a VISA classification for GO. All patients underwent serum thyroid hormone measurement, antibody profile, and 18-fluorodeoxyglucose positron emission tomography/computed tomography (18-FDG PET/CT) of the orbits. The 18-FDG uptake on PET images was expressed in terms of maximum standard uptake value (SUV_max_). CT images were analyzed, and orbital structures were measured in millimeters. Vision, inflammation, strabismus, and overall appearance were assessed according to the VISA classification system, thyroid hormone levels, antibody values, 18-FDG uptake, and thickness of orbital structures.

**Results:**

Altogether, 32 eyes of 16 patients (10 women, 6 men; mean age 44.31 ± 13 years, range 20–71 years) were included. Three patients were hypothyroid, seven were euthyroid, and six were hyperthyroid. CT measurements of extraocular muscle diameter were elevated (*P* < 0.05), and muscle 18-FDG uptake values were increased. Eyes with a clinical VISA inflammation score of ≤ 4 had an average extraocular muscle SUV_max_ of 3.09, and those with a score of ≥ 5 had an average SUV_max_ of 3.92 (*P* = 0.09), showing no clear correlation between clinically observed inflammation and 18-FDG uptake. 18-FDG uptake values also did not show a correlation with extraocular muscle diameter as measured by CT (R^2^ = 0.0755, *P* > 0.05).

**Conclusions:**

We demonstrated a lack of correlation between 18-FDG extraocular muscle uptake and either clinical inflammation score or muscle diameter. Although 18-FDG uptake has been used as an inflammation marker in other pathologies, inflammation in GO may be clinically detected in PET/CT-negative cases, and cases with negative clinical findings may show inflammation on PET/CT. Clinical evaluation is mandatory but may be insufficient and inaccurate for classifying GO. A larger and homogeneous sample size and further research is needed to define the role of PET/CT in detecting, grading, and follow-up of GO to optimize treatment of the inflammatory stage respect clinical methods currently used.

## Background

Graves disease is one of the most common autoimmune illnesses, with an annual incidence in women of 1 in 1000 [1]. In addition to thyroid involvement, ophthalmopathy develops in 25% to 50% of patients [2]. The annual incidence of Graves ophthalmopathy (GO) is 16 in every 100 000 women and 3 in every 100 000 men [3]. Approximately 5% of cases are severe, with chemosis, ptosis, and visual disturbances [1].

Physiopathologic theories of the etiology of GO have pointed to inflammation of the extraocular muscles and connective tissue caused by immunoreactivity of the thyroid-stimulating hormone (TSH) receptors [1,4]. Inflammation and lymphocytic infiltration of fibroblasts are thought to be caused by the presence of anti-TSH receptor antibodies and anti-insulin growth factor receptor 1 (IGF-1R) [1,5].

The expansion of the orbital tissues shifts the eyeball forward, compromising venous outflow. These changes, combined with local production of cytokines and other inflammatory mediators, result in pain, proptosis, periorbital edema, conjunctival injection, and chemosis [1]. The anatomical variability of the orbits and the complex interaction of pathogenic mechanisms pose a challenge to the construction of a reproducible classification system to ensure prompt diagnosis, staging, and treatment [6]. For this reason clinical diagnosis is often augmented with imaging studies. Diagnostic imaging methods such as CT show increased volume in the affected extraocular muscles (EOMs) and in the orbital fat, together or separately. It has been found that the EOM findings usually develop somewhat later in the course of the disease [1]. Assessment of the inflammatory process that causes these abnormalities requires a functional and structural study capable of detecting early asymptomatic orbital abnormalities to provide early treatment and limit the effects of GO [1].

New CT technology allows a detailed evaluation of the orbit in less time, with fewer artifacts, subjecting the patient to less radiation exposure, and with greater spatial resolution [7]. Positron emission tomography (PET) is a non-invasive diagnostic method that is used in the diagnosis and staging of malignancies and is helpful in assessing some forms of inflammation [8]. PET allows functional and metabolic assessment independent of any structural alteration [7]. One of its main advantages is its ability to detect early inflammation prior to structural changes in the tissue [8]. 18-FDG is a radiolabeled analogue to glucose that is used as a quantitative metabolic marker of glucose uptake, which is often increased in malignancies and inflammation [9,10]. Lymphocytes display high affinity for FDG [11]–[13]. Glycolytic metabolism is elevated in oncologic and inflammatory processes.

The objective of this study was to describe imaging findings, both structural and metabolic, by means of PET/CT of patients with GO in different clinical stages.

## Methods

This prospective, observational, cross-sectional study was carried out in the Thyroid Clinic of the “Conde de Valenciana” Ophthalmology Institute. The study was approved by the “Instituto de Oftalmología, Fundación Conde de Valenciana, I.A.P,” and the “Unidad PET/CT Ciclotrón, Universidad Nacional Autónoma de México” Review Board and Ethics Committees and conducted according the tenets of the Declaration of Helsinki. Each subject gave informed consent. We included patients 18 years and older who suffered from GO with some degree of clinical inflammatory activity. Patients either had never been on anti-inflammatory treatment or radiation. Patients underwent a complete ophthalmological evaluation (visual acuity, color and binocular vision, ocular adnexa, eye motility, surface and anterior segment biomicroscopy and fundoscopy) and were assigned a VISA classification [6]. Their serum thyroid hormones and antibody profile were analyzed. 64-Slice CT was performed with the Biograph TruePoint 64 PET-CT scanner (Siemens Medical Solutions, Malvern, PA, USA). A total of 555 MBq (15 mCi) of intravenous 18-FDG was administered, and 2D images were acquired 90 minutes after injection. A transmission CT scan was obtained to correct emission images for photon attenuation. PET-CT was prepared for acquisition and processing with a spatial resolution of 2–5 mm (average 3 mm), with a matrix acquisition of 256, 2.0 zoom, Gaussian filter, FWHM 5.0, 6 iterations, 16 subset, and iterative reconstruction. Orbital CT was acquired with the parameters 380 mAs and 120 kV and the reconstruction values of Kernel H31s medium smooth, slice thickness 3 mm with 3 mm displacement for soft tissue evaluation, homogeneous b20f and b30f filters. Reconstruction of orbital structures was from 0.75 to 1.0 mm. Axial, coronal, sagittal, and volumetric images were generated.

Images were acquired with a protocol of minimal cerebral, visual, and ocular motility stimulation while the patient’s head was immobilized. The patients were instructed to stare at a luminous stimulus.

PET images were analyzed in terms of maximum standard uptake value (SUV_max_), and structures on CT images were analyzed and measured in millimeters. All eyes were divided into two groups according to the VISA classification of inflammation. The first group included all eyes with an inflammation score of ≤ 4, and the second group included all eyes with an inflammation score of ≥ 5. These groups were compared with the SUV_max_ measurements and analyzed to determine whether the correlation of VISA score with SUV_max_ was significant. Clinical, CT, and PET data are expressed as mean values ± SD. Statistical analysis was performed with Student’s *t* test to compare differences between groups. The relation was calculated using Pearson’s correlation. A *P* value of less than 0.05 was considered statistically significant. All statistics in the present study were done using SPSS for Windows (SPSS version 17.0, Chicago, IL).

## Results

We included 32 eyes of 16 patients (10 women, 6 men) with a mean age of 44.31 ± 13 years (range 20–71 years).

The mean time since the diagnosis of hyperthyroidism was 109 months (range 4–420 months) and since the diagnosis of GO was 63 months (0–420 months). Of the 16 patients, two were active smokers, three had suspended tobacco smoking after hyperthyroidism diagnosis, and 11 denied smoking. Nine of the patients had a history of ^131^I administration and 12 patients were under medical treatment for thyroid hormone control. Six of these were treated with methimazole, two with propranolol, and five with levothyroxine. No patients had been treated with anti-inflammatory therapy in the past six months. No patients had been treated with radiation. Three patients were hypothyroid, seven were euthyroid, and six were hyperthyroid despite treatment at the time of the study (Table 1).

**Table 1:** Clinical characteristics

**Patients (n)**	16
**Eyes (n)**	32
**Gender**	
Female, n (%)	10 (62.50)
Male, n (%)	6 (37.50)
**Age (years)**	44.31 ± 13 (20–71)
**Time of diagnosis**	
Hyperthyroidism	109 (4–420)
Graves ophthalmopathy	63 (0–420)
**Smoking**	
Never, n (%)	11 (68.75)
Former, n (%)	3 (18.75)
Active, n (%)	2 (12.50)
^**131**^**I therapy**	
Previous, n (%)	9 (56.25)
Never, n (%)	7 (43.75)
**Thyroid hormonal status**	
Hypothyroid, n (%)	3 (18.75)
Euthyroid, n (%)	7 (43.75)
Hyperthyroid, n (%)	6 (37.50)

### Thyroid status

Serum levels of thyroid hormones ranged widely from normal. The reference values were as follows: T3: 80–220 ng/dL; T4 (thyroxine) 4.5–12.5 mg/dL; TSH 0.4–6.0 U/dL; anti-thyroglobulin antibodies < 150 IU/mL; anti-microsomal antibodies: < 75 IU/mL. The patients’ values were as follows: T3 1.83–467.28 ng/dL; T4 0.99–28.5 mg/dL; TSH 0.02–45.66 U/dL. Two patients had positive anti-thyroglobulin antibody results (> 150 IU/mL), and five patients had positive anti-microsomal antibodies (> 75 IU/mL).

### Ophthalmologic evaluation

The results of the CO-tailored ophthalmologic history and physical examination were divided into four subgroups, according to the VISA classification.

#### Vision

Most patients had a good corrected visual acuity close to 20/20 (mean 20/22.65, range 20/20 to 20/40) with the Snellen letter chart, 0.054 mean logMAR value. Ten eyes had some degree of dyschromatopsia evaluated with the Ishihara chart. No eye showed afferent pupillary defect or optic disc hyperemia.

#### Inflammation

In all, 18 eyes were affected by retro-ocular pain; 21 experienced morning eyelid edema; 15 showed some degree of chemosis; 27 had conjunctival injection; 18 had palpebral injection; 21 had eyelid edema; and 5 had caruncular edema (this variable is not included in the VISA classification).

#### Strabismus

Seven patients suffered from binocular diplopia. Seven patients exhibited compensatory head positioning, and 14 eyes displayed restriction of movements.

#### Appearance

Appearance was evaluated through variables described in the VISA classification and evaluation sheet. It was graded as mild, moderate, or severe. Altogether, 11 eyes were graded mild, 18 moderate, and 3 severe. The exophthalmometry measurements were 17–27 mm in women and 17–23 mm in men*.*

The comprehensive score of the four components of the VISA classification yielded the following results: 10 eyes had some degree of dyschromatopsia. Twenty-one eyes had a score between 1 and 4, and eleven eyes had a score between 5 and 8. Seven patients presented with diplopia, and fourteen eyes showed some restrictions of eye movements, with an average strabismus score of 0.81 ± 1.06 (range 0–3) in the case of diplopia and restriction of eye movements (Table 2).

**Table 2:** VISA clinical characteristics

**Vision (optic neuropathy)**	
Present n (%)	10 (31.25)
Absent n (%)	22 (68.75)
**Inflammation (VISA scale)**	**3.43 ± 2.01 SD**
0 Points, n (%)	0 (0)
1-4 Points, n (%)	21 (65.62)
5-8 Points, n (%)	11 (34.37)
**Strabismus, diplopia, and restriction (0 a 3)**	**0.81 ± 1.06 SD**
Binocular diplopia, n (%)	7 (43.75)
Restriction of eye movements, n (%)	14 (43.75)
**Appearance**	
Mild, n (%)	11 (34.37)
Moderate, n (%)	18 (56.25)
Severe, n (%)	3 (9.37)

### Computed tomography

The extraocular muscle thickness, optic nerve, and superior ophthalmic vein were measured at their maximum diameter in the coronal plane. The medial rectus (MR) thickness range was 2.4–9.5 mm; the superior rectus (SR) 2.6–10.0 mm; the lateral rectus (LR) 2.0–5.9 mm; the inferior rectus (IR) 2.0–9.5 mm; the superior oblique (SO) 2.0–5.5 mm; the inferior oblique (IO) 2.6–6.8 mm; the optic nerve (ON) 2.4–6.5 mm; and the superior ophthalmic vein (SOV) 1.0–3.1 mm (Table 3).

**Table 3:** Muscle/ON/SOV thickness values on CT and uptake on PET

**Structure**	**CT (mm)**		**PET (SUV**_**max**_**)**	
	**Average ± 2 SD**	**Range**	**Average ± 2 SD**	**Range**
**MR**	4.45 ± 1.74	2.4-9.5	3.52 ± 1.44	2.07-4.96
**SR**	5.07 ± 1.94	2.6-10	3.36 ± 1.23	2.13-4.59
**LR**	3.44 ± 0.93\	2-5.9	3.04 ± 1.55	1.49-4.58
**IR**	5.13 ± 1.72	2-9.5	3.50 ± 1.71	1.80-5.21
**SO**	3.11 ± 0.85	2-5.5	3.63 ± 1.23	2.40-4.86
**IO**	4.10 ± 1.26	2.6-6.8	2.59 ± 1.48	1.11-4.08
**ON**	4.08 ± 0.93	2.4-6.5	1.62 ± 0.48	1.14-2.10
**SOV**	1.96 ± 0.59	1-3.1	0	0

*CT* Computed Tomography, *PET* Positron Emission Tomography, *SD* Standard Deviation, *mm* millimeters, *SUV*_*max*_ maximum Standardized Uptake Value, *MR* Medial Rectus, *SR* Superior Rectus, *LR* Lateral Rectus, *IR* Inferior Rectus, *SO* Superior Oblique, *IO* Inferior Oblique, *ON* Optic Nerve, *SOV* Superior Ophthalmic Vein.

### Positron emission tomography

The MR SUV_max_ range was 1.0–7.5; the SR 1.3–5.8; the LR 0.9–6.7; the IR 1.1–8.2; the SO 1.6–6.1; the IO 1.3–5.8; and the ON 1.0–3.1 (Table 3).

The patient with the greatest uptake in the EOMs had a mean SUV_max_ value of 6.71. This correlated with the thickest average measurement obtained by CT, with a mean value of 5.01 mm. A similar condition occurred in another patient, whose values were 4.20 SUV_max_ and 4.19 mm, respectively. This relationship prevailed in only these two patients; the rest had no correlation between uptake and muscle thickness values (R^2^ = 0.0755, *P* > 0.05) (Table 4).

**Table 4:** Maximum uptake and thickness values of extraocular muscles

**Patient**	**FDG uptake**	**Thickness**
	**on PET (SUV**_**max**_**)**	**on CT (mm)**
1	4.20	4.19
2	6.71	5.01
3	2.58	4.40
4	2.35	4.08
5	3.04	4.26
6	3.63	3.73
7	3.35	3.77
8	2.32	3.54
9	1.18	3.39
10	2.73	3.03
11	3.84	3.81
12	5.45	2.87
13	3.43	2.47
14	3.94	3.96
15	2.83	2.65
16	2.44	3.83

*FDG *18 F-fluorodeoxyglucose, *PET* Positron Emission Tomography, *SUV*_*max*_ maximum Standardized Uptake Value, *mm* millimeters.

### PET correlation and clinical inflammation by means of VISA

All eyes were divided into two groups according to the degree of clinical inflammation assigned by the VISA classification. The first group included all eyes with an inflammation score of ≤ 4, and the second group included all eyes with an inflammation score of ≥ 5. These groups were evaluated according the mean value of FDG uptake of EOMs on PET and compared to determine whether the difference in the value of FDG uptake was statistically significant. The first group had a mean SUV_max_ of 3.09, and the second had a value of 3.92 (*P* = 0.09, power 3.91) (Table 5).

**Table 5:** Correlation between inflammation VISA score and average FDG uptake by PET

**Inflammation index (VISA)**	**Averaged SUV**_**max**_	**SD**	**N**
≤ 4	3.09	1.13	21
≥ 5	3.92	1.52	11

*FDG* 18 F-fluorodeoxyglucose, *PET* Positron Emission Tomography, *SUV*_*max*_ maximum Standardized Uptake Value, *SD* Standard Deviation.

### PET/CT correlation

**Figure 1:**
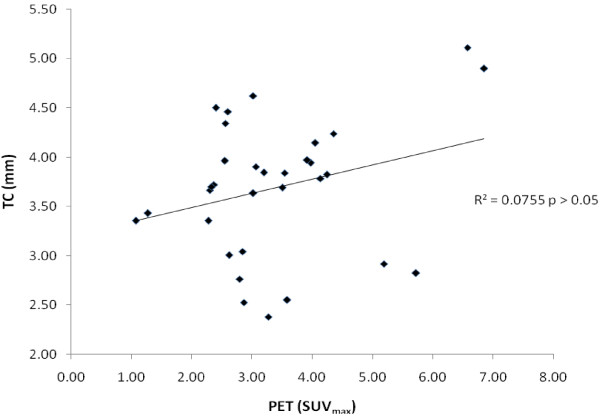
**PET-CT correlation.** PET values showed no correlation with CT measurements according to Pearson correlation results (R^2^ = 0.0755, *P* > 0.05).

There was no statistical correlation of SUV_max_ with CT measurements in the scatterplot when Pearson’s correlation was calculated (R^2^ = 0.0755, *P* > 0.05) (Figure 1).

## Discussion

In 2006, Kuo et al [14] were the first to describe a PET/CT study in a patient with GO. In this case, the detection of inflammation by FDG uptake was demonstrable by this quantitative imaging method. In this study we screened 16 patients (32 eyes) with a GO diagnosis, with a predominance of females (ratio 1.0:0.6) with a wide range of hormone levels.

No patient had a visual acuity lower than 20/40. However, 10 of 32 eyes showed some degree of dyschromatopsia. None presented with an afferent pupillary defect or alterations in the color of the papilla.

Inflammation is one of the most difficult variables to identify clinically due to the variability of its manifestations and because there are many confounding variables, such as noninflammatory fibrosis, in the disorder. Among the signs of inflammation in this study, conjunctival injection was the most common, followed by eyelid edema. Caruncular edema (a sign not included in the VISA classification) was the least frequent. Strabismus was more common than expected. Seven patients showed some degree of binocular diplopia, which made them adopt a compensatory head position, and 14 eyes had some restriction of eye movements. In terms of overall appearance, 18 eyes showed a moderate degree of abnormality.

**Figure 2:**
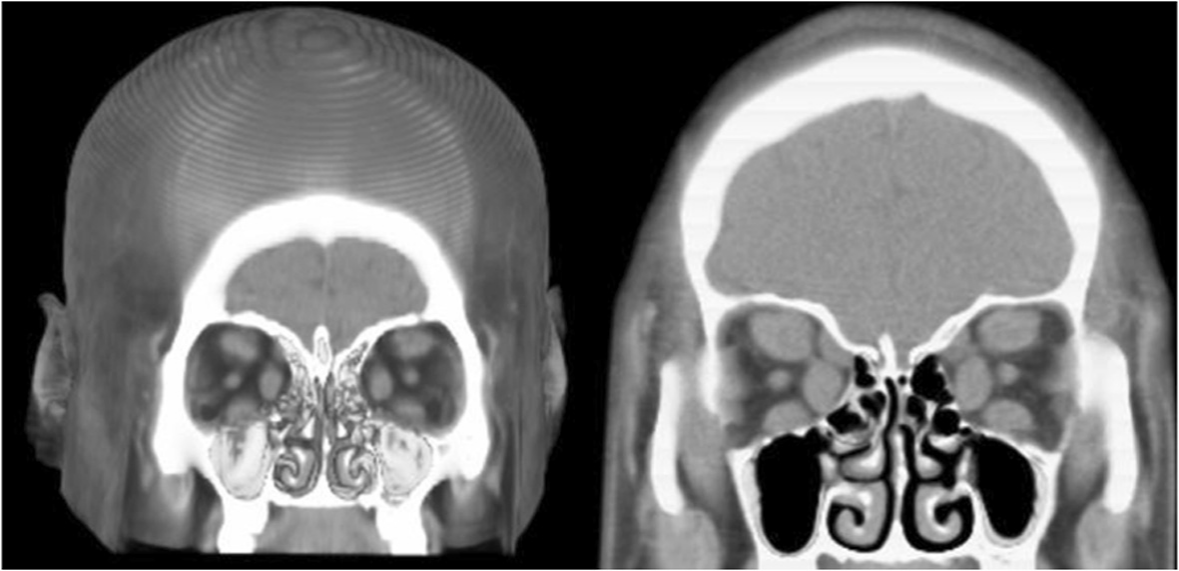
**The thickening of extraocular muscles was evaluated on multislice CT with volumetric reconstruction of a coronal slice.** Left: thickening is evident in the inferior, medial, and superior rectus muscles. Right: thickening of the four rectus muscles and SO.

In 2007, Lerdlum et al [15] described the thickness of the extraocular muscles in a population of 200 people by means of CT, reporting the following average values and ranges: 3.7 mm (2.8–4.6 mm) for the MR, 3.6 mm (2.4–4.8 mm ) for the LR, 3.8 mm (2.4–5.3 mm) for the superior complex (SC), and 4 mm (2.6–5.4 mm) for the IR. Other authors, such as Lee, Ozgene, and Nugent [16]–[18] have reported similar values. In this study, thickness averaged higher than that reported in healthy subjects: 4.45 mm (2.4–9.5 mm) for MR, 3.44 mm (2–5.9 mm) for the LR, 5.07 mm (2.6–10 mm) for the SR, and 5.13 mm (2.0–9.5 mm) for IR. The difference between the thickness of rectus EOMs in healthy individuals according to Lerdlum et al [15],. compared with patients with GO who participated in this study, was statistically significant (*P* < 0.05) in the case of MR, SR/SC, and IR but not for the LR. This is a finding that matches the frequency of involvement of the extraocular muscles in GO according to previous reports [19] (Figure 2, Table 3).

**Figure 3:**
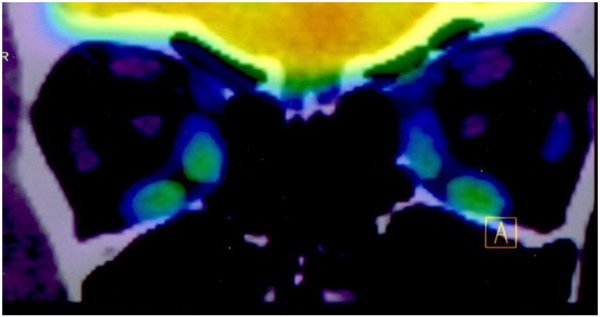
Increased FDG uptake by the inferior and medial rectus muscles on PET/CT.

PET/CT showed increased FDG uptake in the MR and IR (SUV_max_ 3.52 and 3.50, respectively) (Figure 3), which are muscles that are usually thicker in patients with GO [19]. This relationship suggests a difference in timing of the diagnosis, where inflammation precedes muscle thickening and fibrosis. An interesting finding is that the SO muscle showed greater uptake than the rest of the muscles, with an SUV_max_ of 3.63. This does not correlate with the increased muscle thickness in the CT evaluation.

We did not observe a statistically significant difference (*P* = 0.09) in the degree of inflammation assessed clinically by means of the VISA classification and FDG-PET, although the power and the size of the sample in this study is not enough to conclude that there is zero correlation.

There is no gold standard for detecting active inflammation in GO, and VISA is insufficient for this purpose. This suggests that 18-FDG may detect those cases in which clinical assessment is confusing and VISA is not precise. As previously published by our group [20], patients with GO do have increased FDG uptake compared with patients without GO.

Despite the fact that muscular thickness values were equally high in two of the three patients with the highest FDG uptake (Table 4), the PET/CT results showed no correlation (R^2^ = 0.0755, *P* > 0.05), which suggests that the morphological changes are completely independent of the degree of inflammatory activity. Morphological study is useful for evaluating orbital anatomy, but according to our study it may not determine the inflammatory status of the orbital structures.

This study suggests that FDG-PET/CT may be a useful tool for detecting inflammation in GO. The lack of a gold standard tool to detect active inflammation makes it difficult to compare this method and show a statistical difference. VISA may detect inflammation in some cases, but there may be a portion of patients that cannot be correctly diagnosed. It is here that PET/CT may be useful. 18-FDG PET/CT is possibly superior to other imaging studies such as CT alone and even magnetic resonance imaging for detecting active inflammation, as previous studies have reported in other structures [21]. It may be useful for assessing the inflammatory status when clinical doubt exists.

The limitations of this study are the small sample, the different times of onset of the hyperthyroidism and ophthalmopathy, and the differences of hormonal status among the patients.

In conclusion, GO is a pathological entity whose etiology, pathophysiology, epidemiology, clinical assessment, diagnosis, and treatment are under investigation. None of the above aspects has been clarified and understood entirely. Currently, there is no accurate, objective tool for its clinical evaluation and for differentiating active inflammatory states from fibrosis. The study of GO by PET/CT provides valuable and useful information for diagnosis, characterization, and therapy in cases where clinical doubt exists. Further research is required to define the role of FDG-PET/CT in the detection, clinical correlation, classification, and monitoring of GO.

## Abbreviations

PET: Positron emission tomography; CT: Computed tomography; GO: Graves ophthalmopathy; 18-FDG: 18-Fluorodeoxiglucose; SUVmax: Maximum standardized uptake value; VISA: Vision, inflammation, strabismus and appearance; TSH: Thyroid-stimulating hormone; IGF-1R: Insulin growth factor receptor; EOMs: Extraocular muscles; MR: Medial rectus; SR: Superior rectus; LR: Lateral rectus; IR: Inferior rectus; SO: Superior oblique; IO: Inferior oblique; ON: Optic nerve; SOV: Superior ophthalmic vein; SD: Standard deviation.

## Competing interests

The authors declare that they have no competing interests.

## Authors’ contributions

LGRC had the original study idea, have made substantial contributions to conception and design, was involved in all acquisition data, analysis and interpretation. Also was involved in drafting the manuscript and revising it critically for important intellectual content. GAO was involved in all acquisition data, analysis and interpretation. GMV was involved in all acquisition data, analysis and interpretation. EAR have made substantial contributions to conception and design. JLTV have made substantial contributions to conception and design. Also was involved in revising it critically for important intellectual content and have given final approval of the version to be published. All authors read and approved the final manuscript.
